# Whole-Body Composition Features by Computed Tomography in Ovarian Cancer: Pilot Data on Survival Correlations

**DOI:** 10.3390/cancers15092602

**Published:** 2023-05-04

**Authors:** Giorgio Raia, Maria Del Grande, Ilaria Colombo, Marta Nerone, Lucia Manganaro, Maria Luisa Gasparri, Andrea Papadia, Filippo Del Grande, Stefania Rizzo

**Affiliations:** 1 Istituto di Imaging della Svizzera Italiana (IIMSI), Ente Ospedaliero Cantonale, 6900 Lugano, Switzerland; giorgio.raia@eoc.ch (G.R.); filippo.delgrande@eoc.ch (F.D.G.); 2 Service of Medical Oncology, Oncology Institute of Southern Switzerland, Ente Ospedaliero Cantonale (EOC), 6500 Bellinzona, Switzerland; maria.delgrande@eoc.ch (M.D.G.); ilaria.colombo@eoc.ch (I.C.); marta.nerone@eoc.ch (M.N.); 3 Department of Radiological, Oncological and Pathological Sciences, University of Rome Sapienza (IT), 00185 Roma, Italy; lucia.manganaro@uniroma1.it; 4 Department of Gynecology and Obstetrics, Ente Ospedaliero Cantonale of Lugano (EOC), 6900 Lugano, Switzerland; marialuisa.gasparri@eoc.ch (M.L.G.); andrea.papadia@eoc.ch (A.P.); 5 Facoltà di Scienze Biomediche, Università della Svizzera Italiana, 6900 Lugano, Switzerland

**Keywords:** ovarian cancer, body composition, adipose tissue, muscle, bone, calcium

## Abstract

**Simple Summary:**

Five-year overall survival in patients with epithelial ovarian cancer is still set at 50%, going down to 31% in patients presenting with advanced stage disease. Recent studies have shown associations between some body composition values, such as sarcopenia, and overall survival in ovarian cancer patients, but the results are still conflicting. The main aim of this retrospective exploratory study was to assess the associations between many body composition components, extracted from computed tomography performed in routine clinical practice, and overall survival and progression-free survival. Extended body composition evaluation confirmed an association between body composition components (skeletal muscle volume) and overall survival and progression-free survival, as well as associations between intramuscular adipose tissue, epicardial adipose tissue and paracardial adipose tissue with progression-free survival. These promising results encourage further studies assessing the role of body composition in outcomes of ovarian cancer patients.

**Abstract:**

Background: The primary objective of this study was to assess the associations of computed tomography (CT)-based whole-body composition values with overall survival (OS) and progression-free survival (PFS) in epithelial ovarian cancer (EOC) patients. The secondary objective was the association of body composition with chemotherapy-related toxicity. Methods: Thirty-four patients (median age 64.9 years; interquartile range 55.4–75.4) with EOC and thorax and abdomen CT scans were included. Clinical data recorded: age; weight; height; stage; chemotherapy-related toxicity; and date of last contact, progression and death. Automatic extraction of body composition values was performed by dedicated software. Sarcopenia was defined according to predefined cutoffs. Statistical analysis included univariate tests to investigate associations of sarcopenia and body composition with chemotoxicity. Association of body composition parameters and OS/PFS was evaluated by log-rank test and Cox proportional hazard model. Multivariate models were adjusted for FIGO stage and/or age at diagnosis. Results: We found significant associations of skeletal muscle volume with OS (*p* = 0.04) and PFS (*p* = 0.04); intramuscular fat volume with PFS (*p* = 0.03); and visceral adipose tissue, epicardial and paracardial fat with PFS (*p* = 0.04, 0.01 and 0.02, respectively). We found no significant associations between body composition parameters and chemotherapy-related toxicity. Conclusions: In this exploratory study, we found significant associations of whole-body composition parameters with OS and PFS. These results open a window to the possibility to perform body composition profiling without approximate estimations.

## 1. Introduction

Epithelial ovarian cancer (EOC) is the fourth cause of cancer death in the female population in developed countries, with 19,710 estimated new cases and 13,270 estimated deaths in the United States in 2023 [1]. The current standard treatment for EOC is primary cytoreductive surgery with complete resection of all macroscopic disease, followed by adjuvant platinum-based chemotherapy with or without the antiangiogenic agent bevacizumab [2]. When the disease distribution is considered not sufficiently amenable to complete primary cytoreduction, or the patient is not suitable for upfront surgery (inoperable), surgery can be preceded by neoadjuvant chemotherapy (NACT) [3]. Recent advancements in the understanding of EOC biology have led to the incorporation of poly(ADP-ribose) inhibitors (PARP-inhibitors) in the first-line maintenance treatment of patients with stage III–IV EOC, with significant improvement in progression-free survival and overall survival, particularly in patients with *BRCA1* and *BRCA2* mutations or when the tumor has homologous recombination deficiency [3,4,5,6]. However, considering all races, the 5-year overall survival rate is still set at 50%, going down to 31% in patients presenting in advanced stages [1].

Imaging examinations, including computed tomography (CT), represent a regular part of the standard management of preoperative evaluation and follow-up in many cancer patients [7,8,9,10,11], including EOC. CT is also considered a referral method for noninvasive assessment of muscle quantity and distribution of adipose tissue [12,13]. Indeed, many dedicated software programs allow the extraction of quantitative features from a single axial CT image, usually at the level of the third lumbar vertebra (L3), including skeletal muscle area (SMA), subcutaneous adipose tissue (SAT), skeletal muscle density (SMD) and visceral adipose tissue (VAT).

Patients fit for surgery and chemotherapy are expected to show a better prognosis, having no contra-indications for upfront surgery, nor evident contra-indications for full-dose chemotherapy; however, this is not always true, and therefore whether or not body composition profiling at diagnosis is relevant for survival is still an open question. To this end, many studies have assessed the associations between body composition and prognosis in different cancer types [14,15,16,17,18]. Among the studies analyzing EOC [19,20,21,22,23,24,25], the results are conflicting, with some showing an association of overall survival and sarcopenia and others not confirming this association [26]. The limitations of these studies depend mainly on the differences in body composition values evaluated, as well as on the techniques used to extract them and the cut-offs used for classification [26,27].

However, in all the above-mentioned papers, the body composition values were extracted from a single axial CT image and then approximated for the whole body. Nowadays, more recent and advanced software programs allow an automatic assessment of the body composition profiling from the entire CT scan, thus offering a direct and complete body composition evaluation without the need for approximate estimates.

The primary objective of this pilot explorative study was, therefore, to assess associations of automatic whole body composition values extracted from routinely performed CT scans with overall survival (OS) and progression-free survival (PFS) in EOC patients. The secondary objective was to evaluate the association of body composition values with chemotherapy-related toxicity.

## 2. Materials and Methods

### 2.1. Patient Selection

This retrospective study was approved by the Local Ethics Committee with waiver of dedicated informed content. From a database of patients with newly diagnosed EOC, referred to our institution between February 2011 and March 2020, we selected only patients with a pretreatment CT scan including both thorax and abdomen in one acquisition after injection of contrast medium [25].

The main inclusion criteria were age ≥ 18 years, diagnosis of EOC and a CT scan performed within 30 days before starting chemotherapy. The main exclusion criteria were a CT scan of the thorax and abdomen performed more than 30 days before surgery; previous or concurrent malignancy; and technical problems on the CT images, such as from metallic prostheses [28].

### 2.2. Clinical Data Recorded

Age at diagnosis; weight and height to calculate the body mass index (BMI); International Federation of Gynecology and Obstetrics (FIGO) stage; residual disease at surgery (R), classified as R0 = no residual disease; R1 = residual disease less than 1 cm; and R2 = residual disease larger than 1 cm; and NACT, if any. For the secondary endpoint, chemotherapy-related complications were recorded as a dose reduction in any chemotherapy agent compared with the first cycle; premature discontinuation of chemotherapy due to toxicity; and cycle delays >2 weeks due to chemotherapy-induced adverse events. Date of last contact, date of progression (according to clinical evaluation or to progressive disease at CT scan) and date of death were also recorded and updated on 15 February 2023 in order to calculate OS and PFS.

### 2.3. Extraction of Whole-Body Composition Features

**Figure 1:**
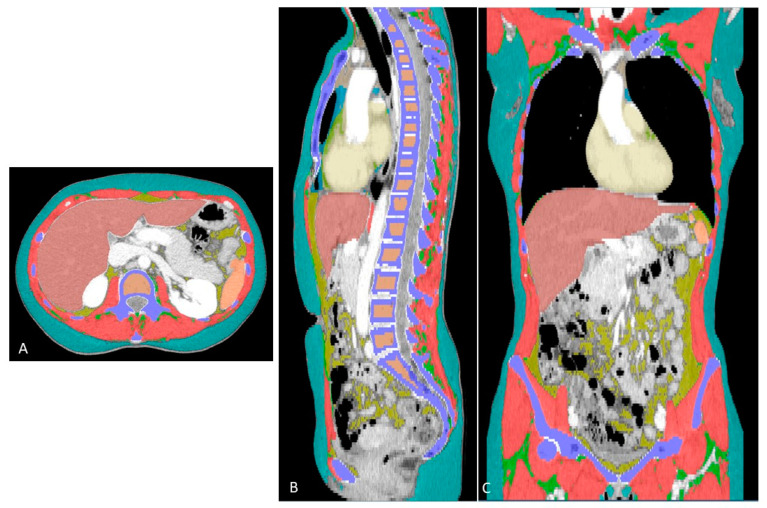
Colored segmentations in the axial (**A**), sagittal (**B**) and coronal (**C**) views. The different colors show each compartment automatically segmented, as follows: red = skeletal muscle; green = intramuscular adipose tissue; cyan = subcutaneous adipose tissue; blue (aqua) = paracardial adipose tissue; green = epicardial adipose tissue; purple = bone; bronze = trabecular bone; brick red = liver; amber = spleen; and ivory = heart.

CT examinations were performed on different CT scanners, 5 from the same vendor (Siemens Healthineers AG, Erlangen, Germany) and 1 from a different vendor (Philips, Eindhoven, The Netherlands). All the acquisition parameters were set homogenously, irrespective of the CT machine. All the CT scans were available in digital format on our picture archiving and communication system. Series used for segmentation were acquired after contrast medium in the portal venous phase. The selected series was uploaded into an input folder to allow a dedicated software program (DAFS, Voronoi Health Analytics Inc., Vancouver, BC, Canada) to curate each individual scan in its own folder. An automatic segmentation engine was enabled for extended body composition analysis of the following specific tissues/organs: skeletal muscle (SKM); intramuscular adipose tissue (IMAT); visceral adipose tissue (VAT); subcutaneous adipose tissue (SAT); visceral and subcutaneous adipose tissue (VAT-U-SAT); epicardial adipose tissue (EpAT); paracardial adipose tissue (PaAT); thoracic adipose tissue (ThAT); bone; trabecular bone tissue (TRBCLR); liver (LIV); spleen (SPL); aortic calcification (AOC); and heart (HRT). The segmentations were visualized and checked in the three planes, as shown in Figure 1.

### 2.4. Statistical Analysis

For continuous variables, median and interquartile range (IQR) were reported, and absolute and relative frequencies were assessed as summary measures of categorical variables. Based on the nature of variables, Fisher’s exact tests and Wilcoxon rank tests were performed to investigate associations between sarcopenia status and parameters of body composition with chemotoxicity. Patients were classified as sarcopenic or nonsarcopenic using the cutoff for SMI [29]. Subsequently, a sensitivity analysis was performed using the median as cut-off.

Progression-free survival (PFS) was calculated from date of surgery to disease progression or death (event), or the last follow-up (censored). Overall survival (OS) was calculated from date of surgery to death (event) or the last follow-up (censored). PFS and OS were estimated with the Kaplan–Meier (KM) method, and survival distributions were compared using log-rank test or univariate Cox proportional hazard model. In the case of continuous variables, the survival area graph was used. This directly extends the standard Kaplan–Meier graph to the continuous case, representing the survival probability as a function of time and a continuous covariate by a color-scaled area [30]. The multivariable Cox proportional hazard model was used to determine the independent prognostic role of sarcopenia and parameters of body composition with cancer progression or death, adjusting for age at diagnosis and/or tumor stage. The significance level was set at a global 2-tailed *p*-value of <0.05 for all analyses. The statistical analyses were performed with Rstudio software, version 4.1.1.

## 3. Results

From a database of 64 patients, 30 were excluded due to a lack of chest CT; therefore, 34 patients met the inclusion and exclusion criteria. As shown in Table 1, the median age at diagnosis was 64.9 (IQR 55.4; 75.4); most patients were diagnosed in stage III (*n* = 20; 58.8%), and 21 patients underwent upfront surgery (61.8%), in most cases with no evident residual disease (R0 = 20; 58.8%).

Median and IQR ranges of all the body composition measurements evaluated are shown in Table 2.

The median follow-up was 35.9 (23.8–40.1) months; the median OS was 35.3 (32.6-NA) months; and the median PFS was 18.1 (11.5–25) months.

**Figure 2:**
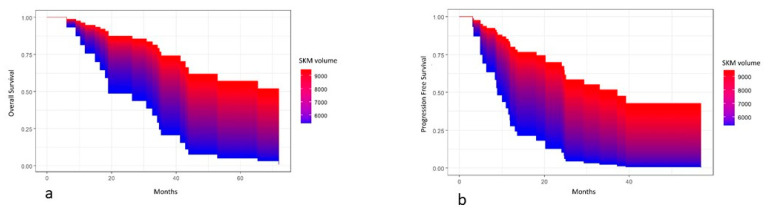
Area graphs showing the association between skeletal muscle (SKM) volume and overall survival (**a**) and progression-free survival (**b**).

The multivariate analysis, adjusting for FIGO stage and age at diagnosis, demostrated a significant association between both SKM volume and OS (*p* = 0.04),with lower values of SKM volume being associated with worse survival, and between SKM volume and PFS (*p* = 0.04), with lower values of SKM volume being associated with shorter PFS (Figure 2).

**Figure 3:**
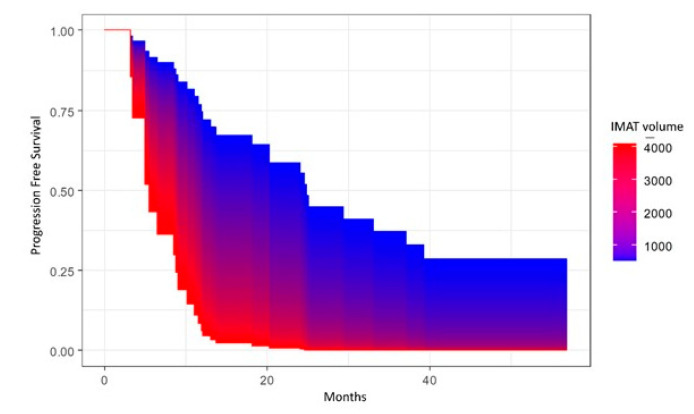
Area graph showing association between intramuscular adipose tissue (IMAT) volume and progression-free survival.

A significant association was also present in the univariate (*p* = 0.03) and multivariate (*p* = 0.049, adjusted for age at diagnosis and FIGO stage) analyses between IMAT volume and PFS, with lower volumes of IMAT being associated with better PFS (Figure 3), whereas this association was not present with OS (*p* = 0.33).

**Figure 4:**
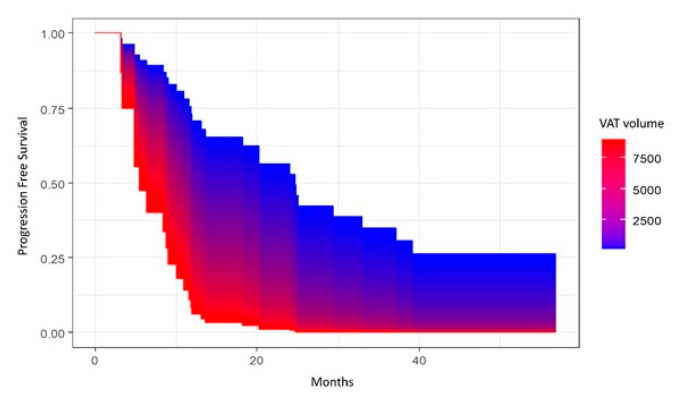
Area graph showing the association between visceral adipose tissue (VAT) volume and progression-free survival.

Univariate and multivariate analyses showed no significant associations between VAT volume and OS. However, there was a significant association in univariate analysis between VAT volume and PFS (*p* = 0.04), with lower volumes of VAT being associated with better PFS (Figure 4), although the *p*-value became *p* = 0.07 when adjusting for age at diagnosis and FIGO stage.

**Figure 5:**
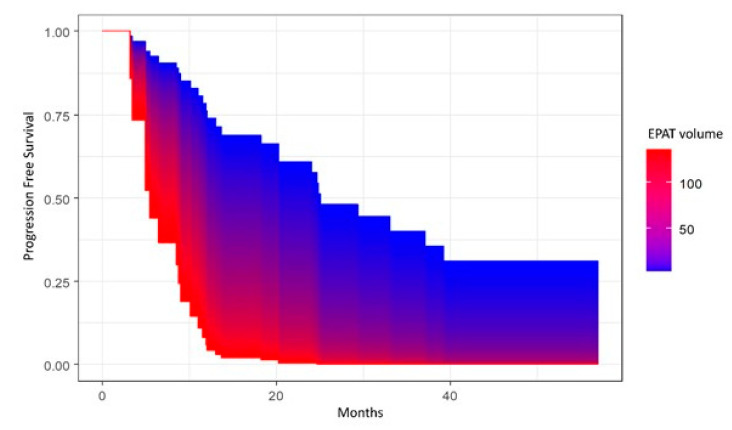
Area graph showing the association between epicardial adipose tissue (EpAT) volume and progression-free survival.

**Figure 6:**
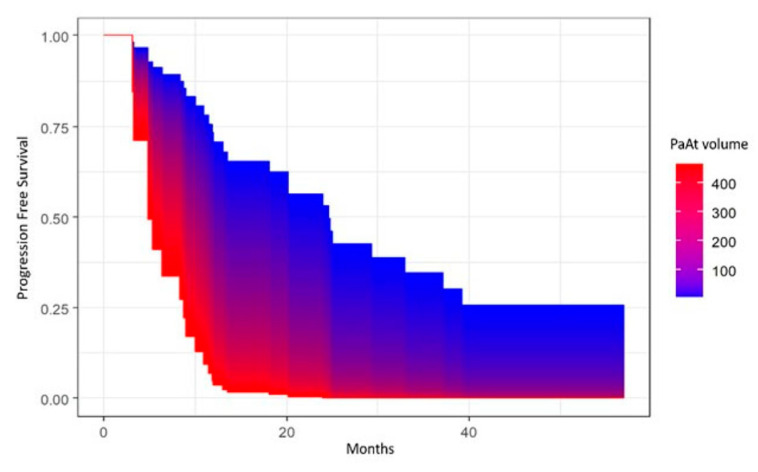
Area graph showing the association between paracardial adipose tissue (PaAT) volume and progression-free survival.

Univariate and multivariate analyses showed a significant association between EpAT and PFS (*p* = 0.01 and *p* = 0.01, respectively), with lower values of EpAT being associated with better PFS (Figure 5), as well as between PaAT and PFS (*p* = 0.02 at univariate analysis and *p* = 0.036, adjusting for age at diagnosis and FIGO stage), as shown in Figure 6, with lower values being associated with better PFS. However, neither EpAT nor PaAT showed significant associations with OS.

Univariate and multivariate analyses did not show significant associations between OS and PFS for volumes of SAT, VAT U SAT, ThAT, LIV, SPL, AOC and HRT.

The analyses dedicated to the secondary endpoints disclosed no associations between the full-body composition parameters and chemotoxicity, in terms of dose reduction, cycle delays, premature termination of chemotherapy and any toxicity. Likewise, sarcopenia, either defined according to the cut-off of Martin et al. [29], or dichotomized according to the median values, did not show associations with OS, PFS and chemotoxicity.

## 4. Discussion

The fundamental abnormality leading to tumor cell growth is the continual unregulated propagation of cancer cells. Despite considerable progress in the treatment of cancer having been achieved with the development of new anticancer drugs, the 5-year survival of OC patients is still as low as 31% for patients in advanced stages, thus suggesting that patient characteristics, such as body composition, may have a role in outcome [31].

Water, fat, proteins (muscle) and minerals (bone), in this order of decreasing amounts, make up the human body. Fat and mass have so far attracted the most attention for health, because it is well established that a high amount of body fat is associated with high morbidity and mortality, although the specific contribution of each individual compartment of adipose tissue is still under debate. Muscle healthiness is a mix of quantity (muscle volume) and quality (strength), while the former can be directly measured by CT imaging (in terms of area or volume), the latter can be indicated by fat infiltration, indicated here by IMAT, also referred to as skeletal muscle density or muscle attenuation in other studies. Sarcopenia, often defined as reduced physical performance following loss of muscle mass, is frequently accompanied by increased fat infiltration in the muscles. Indeed, the infiltration of fat messes up muscle fibers, eventually leading to a loss of strength [32]. Different studies thus refer to sarcopenia as indicated by either a reduction in muscle quantity, or a reduction in muscle quality (high fat infiltration), or both.

In this study, we found a significant association between skeletal muscle volume, directly measured from the entire volume of muscles of thorax and abdomen, with OS and PFS. Indeed, patients with lower SKM showed worse OS and PFS, although sarcopenia, defined by SMI values, did not. This apparent discrepancy between the significance of SMA volume and SMI can depend strictly on what these parameters represent. Indeed, SKM volume as measured in this study is the direct measurement of the entire amount of muscle included in the CT scan, whereas SMI is an indirect measurement of muscle, usually calculated from the muscle area on one single slice of CT divided by the square height. Although Ubachs et al. demonstrated a significant effect of sarcopenia on OS (0.007; HR: 1.11, 95% CI: 1.03–1.20) [24] in their meta-analysis, the inconsistency in the definition of optimal SMI cut-off levels across studies demonstrates some weakness in this method. This is confirmed by the discrepant results of other studies, such as Kim et al., who, in a Korean population, did not demonstrate a significant difference in OS and PFS between sarcopenic and nonsarcopenic patients [33].

As a complementary finding for muscle quality, in our cohort, we found a significant association of high levels of IMAT (representing the fat infiltration of muscle) with worse PFS. These results are concordant with Kumar et al., who demonstrated that lower levels of mean skeletal attenuation were significantly associated with poorer survival [32]. In a cohort of 323 patients, Ataseven et al. demonstrated a significant association between muscle attenuation and poor survival, especially in patients with residual tumor at upfront surgery [34], proposing a cut-off value of less than 32 HU for more frequent associations with poor OS in patients with residual tumor. Interestingly, two studies that demonstrated a significant association between low SMI and OS included a high rate of patients that did not achieve a complete resection rate [35,36], which is still the most important predictor of survival.

A large amount of VAT is a known risk factor for adverse cardiovascular events [37,38]. In our cohort, we demonstrated a significant association between VAT and PFS, with a lower volume of VAT being significantly associated with better PFS. The L3 lumbar vertebra landmark is often used in cross-sectional body composition analysis because it should correspond to whole-body tissue measurements [14]. However, the amount of fat varies according to sex, age and body level. Thus, previous studies suggested that VAT should be calculated by obtaining measurements at several different anatomic levels [39].

One reason that prompted us to undertake this study, although with an exploratory attempt, is the fascinating opportunity to effectively quantify body composition profiling from the entire body, with no estimates that can be affected by body shape, sex, or height. With this in mind, a significant association between VAT volume and PFS confirms that the complexity of fat distribution might merit further studies, possibly assessing the real fat volume of different compartments.

In our pilot study, we found a significant association between EpAT and PaAT and PFS. Epicardial fat is an adipose tissue deposit between the visceral pericardium and the myocardium, without a structure or fascia separating it from the myocardium. The paracardial fat is an adipose tissue accumulation in the mediastinum outside the parietal pericardium [40]. Under physiological conditions, epicardial fat is a source of energy for the myocardium, and it shields against the toxicity of free fatty acids. Epicardial fat increases in states of positive energy balance, when the free fatty acids in the blood are converted into triglycerides and accumulate initially in adipocytes and then in nonfat cells [41]. Epicardial fat thickness, volume and total area can be accurately measured by CT. An independent association between pericardial fat and cardiovascular risk factors has been demonstrated [40,42]. EpAT has been consistently associated with metabolic syndrome and coronary artery disease, although there are inconsistencies in the nomenclature and measurement methods in the various studies [43,44,45]. To date, there are few studies assessing the associations between EpAT and PaAT and pathological conditions other than cardiovascular disease. In this regard, Gaborit et al. studied the association between weight loss and epicardial/pericardial fat in bariatric surgery patients and demonstrated that bariatric surgery significantly reduced epicardial fat volumes, but this was not correlated with a decrease in visceral abdominal fat or BMI, suggesting heterogenous effects of weight loss on different fat depots [46]. More recently, a study focusing on the metabolic effects of paracardial fat remodeling demonstrated that the size and function of this fat deposit are controlled by alcohol dehydrogenase 1, an enzyme that oxidizes retinol into retinaldehyde. Furthermore, metabolomics analysis revealed that paracardial fat controlled the levels of circulating metabolites affecting fatty acid biosynthesis [47]. Although still under evaluation, the abovementioned studies demonstrate that the fat around the heart does have a metabolic role, likely not only in cardiovascular disease, and our results suggest that further evaluations are warranted, even at the preclinical level.

This study has some limitations, the first being the small number of patients included. This is the reason why we consider this an exploratory study. Indeed, we needed to assess the feasibility of evaluating the whole-body composition profiling with a new tool, implying downloading the entire CT scan instead of selecting a single slice to be analyzed, in order to decide whether this type of study may merit further efforts in larger cohorts. Furthermore, given the small sample size, we could neither adjust for other confounding factors so as not to overfit in multivariable models nor conduct stratified analyses, for example, by FIGO stage. Another limitation is that we included patients whose CT scan was acquired at least 3 years ago. However, since the CT technology in our hospital is very up-to-date, we are confident that the examinations included are not affected by technical limitations. Furthermore, this long time period allowed us to have a longer follow-up and to update the events related to prognosis, such as progression and/or death. The automatic segmentation can be considered another limitation. Indeed, it is well known that manual and semiautomatic segmentations are more precise, but they are burdened by low reproducibility, whereas automatic segmentations may be less precise but are always reproducible. However, in this case, automatic segmentation is a real advantage in terms of workflow, because the segmentation of every tissue in each slice would be a real time-consuming process, which is also why a single slice is usually chosen.

## 5. Conclusions

In conclusion, in this exploratory assessment of whole-body composition profiling based on computed tomography, in EOC patients, we found significant associations between skeletal muscle volume and OS and PFS, as well as between intramuscular fat, epicardial fat and paracardial fat and PFS. Our results, although preliminary and needing further exploration, open a window to the concrete possibility of performing direct body composition profiling, with no further need for approximate estimations.

## Figures and Tables

**Table 1:** Patient characteristics (*n* = 34).

	N (%)
Age at diagnosis, median (IQR)	64.9 (55.4;75.4)
FIGO Stage IIB IIIA IIIC IV	1 (2.9) 2 (5.9) 18 (52.9) 13 (38.3)
NACT 0 1	21 (61.8) 13 (38.2)
Outcome R0 (no residual disease) R1 (residual disease < 1 cm) R2 (residual disease > 1 cm) NA	20 (58.8) 7 (20.6) 4 (11.8) 3 (8.8)
BMI, median (IQR)	22.9 (21.7; 26.2)

IQR = interquartile range; FIGO = International Federation of Gynecology and Obstetrics; NACT = neoadjuvant chemotherapy; R = residual disease; NA = not available; BMI = body mass index.

**Table 2:** Body composition values exctracted from the whole computed tomography scan.

Values	Median (IQR)
SKM Volume (mm^3^) HU mean HU standard deviation	7793.3 (6829.1; 8286.4) 46.0 (42.8; 51.9) 30.2 (29.0; 31.4)
IMAT Volume (mm^3^) HU mean HU standard deviation	1143.5 (935.9; 1392.0) −50.77 (−55.3; −47.7) 29.4 (28.3; 31.2)
VAT Volume HU mean HU s standard deviation	1624.6 (942.6; 2028.2) −74.3 (−80.3; −65.0) 24.0 (22.2; 26.2)
SAT Volume (mm^3^) HU mean HU standard deviation	9908.5 (7749.1; 14,534.1) −94.8 (−102.1; −90.5) 21.4 (19.8; 23.5)
VAT U SAT Volume (mm^3^) HU mean HU standard deviation	11,503.6 (8957.5; 17,146.9) −92.1 (−99.6; −87.4) 23.0 (21.9; 25.06)
EpAT Volume (mm^3^) HU mean HU standard deviation	31.4 (15.8; 45.0) −58.0 (−66.5; −49.8) 27.7 (26.5; 30.3)
PaAT Volume (mm^3^) HU mean HU standard deviation	79.1 (46.3; 105.3) −71.9 (−79.5; −61.5) 27.1 (25.9; 28.7)
ThAT Volume (mm^3^) HU mean HU standard deviation	19.7 (12.4; 39.1) −68.7 (−79.6; −61.5) 27.1 (25.2; 28.5)
Bone HU mean HU standard deviation	378.1 (316.5; 429.9) 248.3 (233.8; 303.3)
TRBCLR HU mean HU standard deviation	137.2 (125.1; 166.8) 89.2 (80.3; 94.3)
LIV Volume (mm^3^) HU mean HU standard deviation	1398.0 (1239.8; 1655.2) 118.7 (108.4; 126.8) 22.3 (19.6; 25.2)
SPL Volume (mm^3^) HU mean HU standard deviation	162.7 (123.2; 195.4) 116.5 (105.2; 126.7) 23.9 (19.9; 29.1)
AOC Volume (mm^3^)	0.23 (0.008; 0.82)
HRT Volume (mm^3^)	589.3 (537.3; 651.8)

SKM = skeletal muscle; HU = Hounsfield units; IMAT = intramuscular adipose tissue; VAT = visceral adipose tissue; SAT = subcutaneous adipose tissue; VAT U SAT = visceral adipose tissue and subcutaneous adipose tissue; EpAT = epicardial adipose tissue; PaAT = paracardial adipose tissue; ThAT = thoracic adipose tissue; TRBCLR = trabecular bone; LIV = liver; SPL = spleen; AOC = aortic calcium; HRT = heart.

## Data Availability

Data are not publicly available. They can be requested from the corresponding author who will share the coded data upon specific approval of the Ethics Committee.
